# Ngt-1: A glycosyltransferase that confers resistance to three distinct antibiotic classes

**DOI:** 10.1016/j.jbc.2026.111479

**Published:** 2026-04-22

**Authors:** Amna Abbas, David Sychantha, Kalinka Koteva, Mei Chiao, Akosiererem Sokaribo, Dirk Hackenberger, Sara Andres, Gerard D. Wright

**Affiliations:** 1 Department of Biochemistry and Biomedical Sciences, David Braley Centre for Antibiotic Discovery, M.G. DeGroote Institute for Infectious Disease Research, McMaster University, Hamilton, Ontario, Canada; 2 Department of Chemistry, University of Waterloo, Waterloo, Ontario, Canada

**Keywords:** antibiotics, drug inactivation, enzyme mechanism, glycosyltransferase, resistance

## Abstract

Environmental microbes are a rich source of antibiotic resistance genes with the potential to be mobilized and captured by pathogens under selective pressure. This largely unexplored resistome offers an opportunity to identify novel resistance mechanisms that may eventually emerge in clinical settings, supporting efforts to maintain current antibiotic efficacy. From a screen of environmental bacterial isolates, we identified *Bacillus thuringiensi*s WAC10774 B, which confers resistance to the aminocoumarin antibiotic novobiocin through enzymatic inactivation. Analysis of the inactive antibiotic revealed that WAC10774 B modifies novobiocin *via* glycosylation, a unique transformation not reported since the antibiotic’s discovery 70 years ago. Using activity-guided protein purification, we identified the novobiocin glycosyltransferase, Ngt-1, and its encoding gene. Surprisingly, *ngt-1* confers resistance to three structurally distinct antibiotic classes: novobiocin (aminocoumarin), fidaxomicin (tiacumicin), and salinomycin (polyether). To further explore Ngt-1’s functional breadth, we performed steady-state kinetic and protein-structure studies, revealing Ngt-1 as a novel, broad-spectrum glycosyltransferase capable of inactivating antibiotics. These findings highlight the importance of exploring the environmental resistome to identify new resistance enzymes and to inform strategies to combat antimicrobial resistance.

Antimicrobial resistance represents one of the most pressing challenges to modern medicine. Bacteria can acquire resistance through changes to intrinsic cellular features, including point mutations in antibiotic targets or altered expression of general resistance mechanisms, such as efflux systems. In addition, resistance frequently arises through the acquisition of dedicated enzymes that directly inactivate antibiotics. These specialized enzymes function by either degrading the drug (*e.g.*, hydrolysis of β-lactams) or modifying the drug scaffold *via* acetylation, phosphorylation, and glycosylation, thereby reducing target binding, cellular uptake, or antibiotic activity (1, 2). These modification-based mechanisms are especially interesting, as they can act across structurally diverse compounds and contribute to broad-spectrum or low-level resistance phenotypes. One such example is the AAC(6′)-Ib-cr, a variant of an aminoglycoside-modifying acetyltransferase notable for its expanded substrate specificity – extending from aminoglycosides to structurally unrelated fluoroquinolones (3). These specialized proteins may be chromosomally encoded or carried on mobile genetic elements such as plasmids and transposons, facilitating their dissemination across bacterial populations (4, 5). Although pathogens are most often studied due to their direct impact on treatment outcomes, resistance mechanisms, especially enzyme-mediated resistance (1, 6), are also widespread among non-pathogenic environmental bacteria, where they play important ecological roles (4, 5, 7). Understanding the diversity, activity, and distribution of these enzymes is therefore critical for appreciating their role in the evolution and dissemination of antimicrobial resistance.

Together, natural production and human-derived contamination create persistent selective pressures that favor the evolution, maintenance, and diversification of resistance mechanisms within environmental microbial communities. The prevalence of resistance genes in environmental bacteria is therefore unsurprising, given the ancient origins of antibiotics (8) and the contemporary widespread and sustained use of antibiotics across natural and human-impacted ecosystems. In soil and other natural environments, antibiotic-producing microorganisms (producers) continuously release specialized secondary metabolites as a competitive strategy to shape microbial communities (9, 10). Concurrently, these producers evolve self-resistance mechanisms to protect themselves from such toxic compounds (11). This baseline chemical warfare has been considerably amplified by anthropogenic activities; over the past several decades, global antibiotic consumption has steadily increased due to expanded use in human medicine, livestock production, agriculture, and aquaculture. As a result, large quantities of these compounds enter sewage systems, wastewater treatment plants, agricultural soils, and aquatic environments, where regulation and removal remain incomplete (9, 10). The presence of antibiotics in these settings, originating from both natural producers and anthropogenic use, imposes strong selective pressure on surrounding microbial communities, maintaining intrinsic resistance in non-producers and promoting the amplification and dissemination of resistance genes (12).

In this context, environmental microbes constitute a rich reservoir of intrinsic and acquired resistance elements, including enzymes that modify antibiotics. Indeed, we see that antibiotic resistance elements are widespread even in non-producing bacteria; previous work by our group shows that the ratio of resistant environmental bacteria that do not produce glycopeptide antibiotics to those that do is approximately 10:1 (13). A growing body of genomic evidence, supported by the original biochemical observations by Davies in the early 1970s that self-resistance genes of antibiotic-producing bacteria could be the source of clinical resistance (7), suggests that under selective pressures, environmental resistance genes can be enriched, mobilized on transferable genetic elements, and may ultimately be disseminated into pathogenic bacteria. Furthermore, some proteins provide only low or modest resistance. These are referred to as proto-resistance elements, genes that do not currently cause resistance but have the potential to evolve into clinically relevant resistance determinants (14). Over time, increased expression or mutations that enhance enzyme activity can turn these genes into stronger drivers of resistance (14). In this way, the environmental resistome acts not only as a reservoir of existing resistance mechanisms but also as a source of hidden functions that could evolve into specific and efficient forms of resistance.

Consistent with this perspective, our team's previous work has identified clinically relevant resistance determinants with clear environmental origins. We explored the depth of the environmental resistome through metagenomic analyses of ancient DNA from 30,000-year-old Beringian permafrost, revealing the presence of the vancomycin resistance elements VanH, VanA, and VanX. The work highlighted the structural and functional similarities between VanA and its modern variants in contemporary vancomycin-resistant enterococci. Additionally, we identified a highly diverse collection of genes encoding resistance to β-lactam, tetracycline, and glycopeptide antibiotics, highlighting that resistance predates clinical antibiotic use and originates in the environment (4). In other studies, Poirel *et al.* observed that genes encoding CTX-M extended-spectrum β-lactamases were carried by environmental organisms such as *Kluyvera* before appearing in clinical settings (15). Altogether, a growing body of literature suggests that resistance genes have been mobilized from non-producers into pathogens (16, 17, 18, 19). Proactive exploration of environmental isolates and metagenomes for new resistance elements may therefore provide an ‘early warning system’ to anticipate resistance that may eventually be mobilized into clinically relevant pathogens.

Consistent with the need to better understand enzyme-mediated antibiotic modification in environmental reservoirs, we investigated resistance determinants in environmental microbes isolated from a single soil sample. Through this analysis, we identified a *Bacillus thuringiensis* strain harboring a promiscuous glycosyltransferase, Ngt-1. Biochemical characterization of Ngt-1 demonstrates its ability to inactivate the structurally distinct antibiotics novobiocin, fidaxomicin, and salinomycin through glycosylation. This activity highlights the capacity of glycosyltransferases to function as broad-spectrum resistance enzymes, extending beyond the narrow substrate specificity often associated with modification-based mechanisms (1, 2). Notably, Ngt-1 represents a proto-resistance element with activity spanning multiple antibiotic classes, including aminocoumarins, tiacumicins, and polyethers. These findings underscore the potential for environmentally derived glycosyltransferases to contribute to the evolution and dissemination of antimicrobial resistance and reinforce the importance of exploring the environmental resistome as a source of emergent resistance mechanisms.

## Results

### *Bacillus thuringiensis* WAC10774 B inactivates novobiocin

A screen of diverse antibiotic classes on 200 strains obtained from a single soil sample identified WAC10774 as an isolate that appeared to inactivate novobiocin. Novobiocin is a natural product aminocoumarin antibiotic that binds to the β-subunit of DNA gyrase, an essential Type II topoisomerase required for DNA replication (20). Previous studies showed that resistance to novobiocin occurs primarily through point mutations in GyrB (21), and there have been no reports of inactivating enzymes for this class of antibiotics.

**Figure 1:**
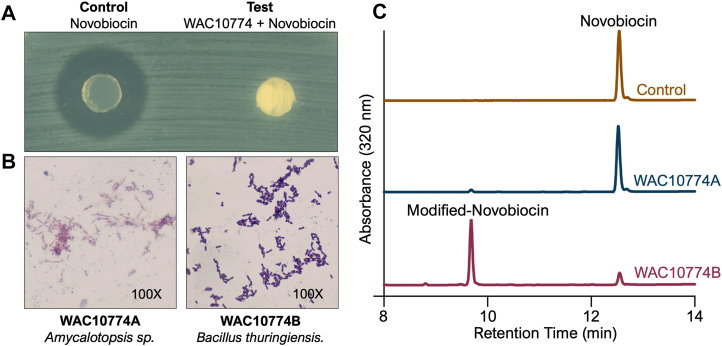
***Bacillus thuringiensis* WAC10774 B modifies novobiocin, resulting in antibiotic inactivation.***A*, a control plug (LB agar + 40 μM novobiocin) placed on a freshly swabbed, novobiocin-sensitive *E. coli ΔbamB ΔtolC* lawn produced a clear zone of inhibition. In contrast, a plug derived from a WAC10774 lawn grown on LB agar + 40 μM novobiocin showed no inhibition zone when applied to the same strain, indicating that WAC10774 inactivates novobiocin. *B*, gram stain of WAC10774 shows the presence of two strains: WAC10774 A – an Amycolatopsis sp., and WAC10774 B – *B. thuringiensis*. *C*, reverse-phase HPLC analysis of culture media from WAC10774 A (*blue*) and WAC10774 B (*red*) whole cells incubated with 50 μM novobiocin compared to the buffer control (*brown*) reveals that WAC10774 B produces a modified product. The novobiocin signal is observed at a retention time of 12.8 min, and the modified novobiocin product is observed at a retention time of 9.7 min.

We assessed novobiocin inactivation using an agar plug assay. In this assay, plugs were taken from control plates containing novobiocin and produced a clear zone of inhibition when placed on a lawn of antibiotic-hypersusceptible *Escherichia coli BW25113 ΔbamB ΔtolC* (22, 23). On the other hand, plugs taken from novobiocin-containing plates incubated with WAC10774 showed a marked reduction in the inhibition zone compared with the control (Fig. 1*A*). This loss of antibiotic activity indicated that WAC10774 reduces the local concentration of novobiocin along the plug, consistent with antibiotic inactivation. To characterize the isolate, we passaged WAC10774 by serial subculturing and determined that it was comprised two distinct strains (Fig. 1*B*). Whole-genome sequencing identified them as *Amycolatopsis* sp. (WAC10774 A, Accession- SAMN57036074) and *B. thuringiensis* (WAC10774 B, Accession- SAMN57016808). To identify the strain responsible for the observed loss of novobiocin activity, we incubated whole cells of each strain with novobiocin and analyzed the conditioned media from the reaction by RP-HPLC. Conditioned media extracted from the *Amycolatopsis* sp. WAC10774 A incubation showed no change compared to control novobiocin, while *B. thuringiensis* WAC10774 B produced a pronounced shift in the novobiocin retention time, supporting chemical modification of the antibiotic consistent with transformation into a more hydrophilic compound (Fig. 1*C*).

### Novobiocin inactivation is a result of glycosylation

**Figure 2:**
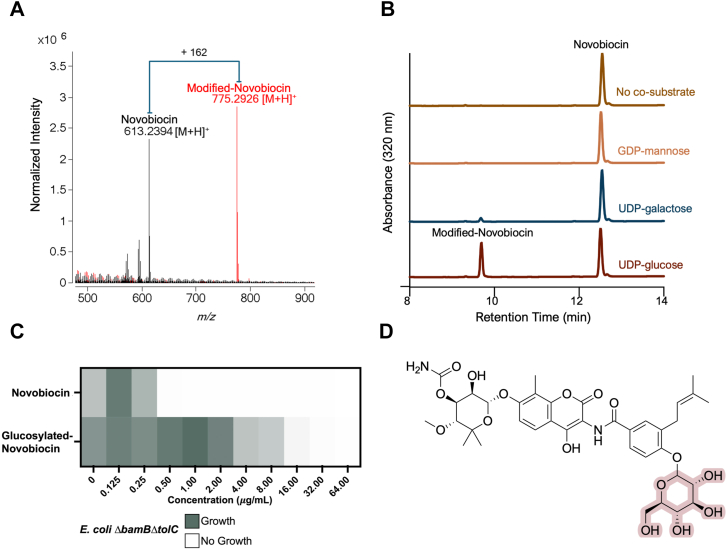
**Novobiocin modification occurs through glycosylation using UDP-glucose as a co-substrate.***A*, liquid chromatography quadrupole time-of-flight mass spectrometry reveals 613.2394 [M + H]^+^ corresponding to the mass of novobiocin and 775.2926 [M + H]^+^ for the modified product. The difference of 162.0532 Da suggests the addition of a hexose group to novobiocin. *B*, RP-HPLC chromatograms testing WAC10774 B cell-free extract supplemented with 50 μM novobiocin in the absence (*brown*) and presence of 250 μM of different sugar donors such as GDP-mannose (*orange*), UDP-galactose (*blue*), and UDP-glucose (*red*). Reaction supplemented with UDP-glucose (*red*) shows a modified novobiocin product at a retention time of 9.7 min, indicating that UDP-glucose is a suitable co-substrate. *C*, minimum inhibitory concentrations heatmap for *E. coli ΔbamB ΔtolC* exposed to novobiocin and glucosylated-novobiocin, demonstrating a 64-fold reduction in antibacterial efficacy following glycosylation. *Gray* indicates growth, while white indicates no growth. *D*, structure of glucosylated-novobiocin; modified at its benzamide hydroxyl.

To understand the molecular basis of novobiocin modification, conditioned media from the previous WAC10774 B reaction was analyzed using liquid chromatography quadrupole time-of-flight mass spectrometry (LC-QToF-MS) to identify post-incubation compounds and their associated masses. Novobiocin has a retention time of 12.8 min (Abs 320 nm) with the predicted m/z value of 613.2394 [M + H]+. The new peak observed following incubation with WAC10774 B showed a retention time of 9.7 min (Abs 320 nm) with an *m/z* value of 775.2926 [M + H]^+^, corresponding to a mass increase of 162.0532 Da, which is consistent with the glycosylation of novobiocin by a hexose moiety (Fig. 2*A*).

To confirm that novobiocin modification was due to glycosylation, cell-free extracts from *B. thuringiensis* were tested for novobiocin modification (50 μM) in the presence of common sugar donors (250 μM): GDP-mannose, UDP-galactose, or UDP-glucose. Only UDP-glucose produced a glucosylated product (Fig. 2*B*).

To assess whether glucosylated novobiocin loses its antibiotic activity, the minimum inhibitory concentration (MIC) of novobiocin and the purified glucosylated novobiocin product were evaluated against the novobiocin-susceptible *E. coli* Δ*bamB* Δ*tolC* strain. This strain exhibits a MIC of 0.5 μg/ml for novobiocin and 32 μg/ml for the glycosylated product (Fig. 2*C*). This 64-fold reduction in antibacterial efficacy confirms that glycosylation renders novobiocin ineffective.

The glucosylated product was further analyzed by tandem mass spectrometry (MS/MS) to determine the position of the glucosyl modification, as three distinct hydroxyl groups in novobiocin could potentially undergo glycosylation. Based on the fragmentation pattern of 775.29*m/z* compared to that of 613.23*m/z*, a peak at 351.14*m/z* was observed, consistent with the benzamide ring modification (Fig. S1). The modification site was confirmed by NMR analysis of the glycosylated product (Table S1). *B. thuringiensis* WAC10774 B therefore glycosylates novobiocin at its benzamide hydroxyl using UDP-glucose as a co-substrate, representing a novel modification of this antibiotic and the first report of enzyme-mediated inactivation of an aminocoumarin antibiotic (Fig. 1*D*).

### *Ngt-1* is a novobiocin resistance gene that also targets three additional antibiotics

To identify the glycosyltransferase responsible for novobiocin modification, we developed a four-step, activity-guided protein purification protocol, coupled with proteomic analysis. Crude whole cell extracts of WAC10774 B were fractionated through ammonium sulfate precipitation, hydrophobic interaction chromatography, hydroxyapatite chromatography, and anion exchange (Table S2, Fig. S2). The semi-pure protein sample was digested with trypsin, and the peptide fragments were analyzed and identified by mass spectrometry. *De novo* peptide mapping was conducted using FragPipe, powered by MSFragger. The peptide fragments were compared against the *B. thuringiensis* proteome to identify proteins present in the semi-pure protein sample (24). A protein sequence annotated as a glycosyltransferase was identified as a potential candidate from that list. To validate its role in novobiocin resistance, the corresponding gene, which we named *ngt-1*, was cloned into the high-copy vector pGDP1 (23), under control of a constitutive P_bla_ promoter and introduced into *E. coli* Δ*bamB* Δ*tolC*. In contrast to the empty-vector control, *E. coli* Δ*bamB* Δ*tolC* containing the *ngt-1* gene showed a 256-fold increase in the novobiocin MIC, indicating that *ngt-1* alone confers resistance (Table 1). We named this enzyme novobiocin glycosyltransferase-1 (Ngt-1).

**Table 1:** Minimum inhibitory concentrations (MIC) in the presence and absence of *ngt-1* gene

Antibiotic	*E. coli* BW25113 *ΔbamB ΔtolC* MIC (μg/ml)	*E. coli* BW25113 *ΔbamB ΔtolC*+*ngt-1* MIC (μg/ml)
Novobiocin	0.5	128
Fidaxomicin	16	128
Salinomycin	4	32

**Figure 3:**
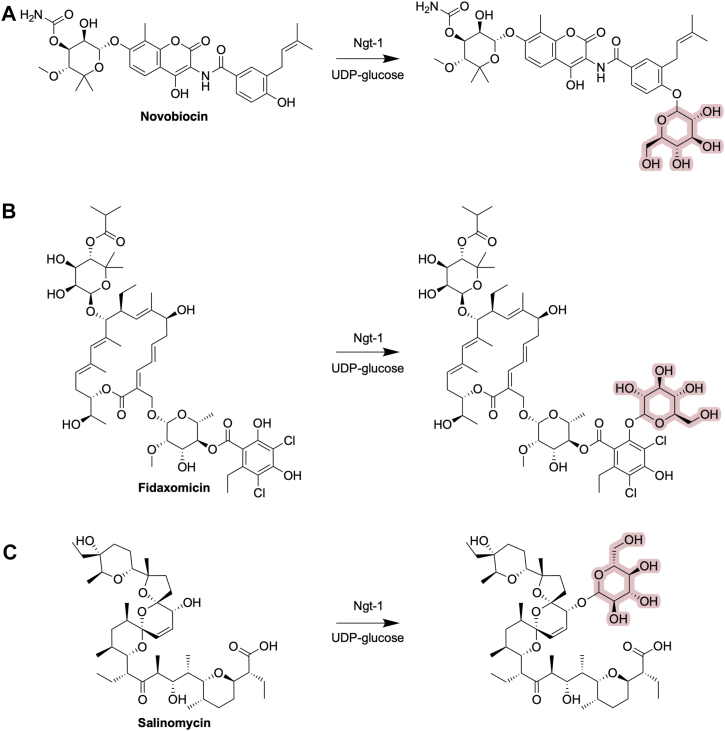
**Modification sites of Ngt-1 targeting antibiotics.** The addition of a glucose group on (*A*) novobiocin, (*B*) fidaxomicin, and (*C*) salinomycin, displaying the sites of modification by Ngt-1, is highlighted in *pink*.

RT-qPCR analysis on the host *Bacillus* strain showed that *ngt-*1 was not inducible in the presence of novobiocin. We considered that, due to the nature of constitutive expression of *ngt-1* in the host, Ngt-1 may accept other antibiotics as substrates for modification. Indeed, additional testing against a panel of antibiotics (Table S3) revealed that *ngt-*1 confers resistance to two additional antibiotics, fidaxomicin (tiacumicin) and salinomycin (polyether), alongside novobiocin (aminocoumarin) (Table 1). Tandem MS/MS was used to predict the site of modification of these antibiotics (Fig. 3).

### Steady-state kinetic analysis of Ngt-1

**Figure 4:**
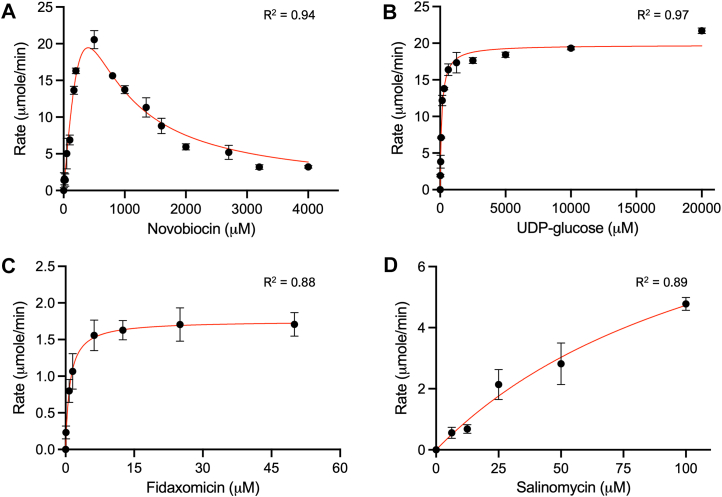
**Steady-state kinetics for Ngt-1.***In vitro* kinetics for Ngt-1 with (*A*) novobiocin, (*B*) UDP-glucose, (*C*) fidaxomicin, and (*D*) salinomycin. Ngt-1 shows substrate inhibition with novobiocin (*A*), while exhibiting Michaelis-Menten kinetics with UDP-glucose (*B*) and fidaxomicin (*C*) upon saturation.

Having identified *ngt-1* as a gene encoding a multi-antibiotic modifying enzyme, we next sought to characterize how Ngt-1 interacts with its antibiotic substrates through steady-state kinetic analysis. The soluble and active N-terminal His_6_-tagged Ngt-1 was successfully overexpressed in *E. coli* BL21 and purified. Steady-state kinetic studies of Ngt-1 were conducted, monitoring ADP release using a pyruvate kinase-lactate dehydrogenase coupled assay (25). Ngt-1 exhibited substrate inhibition with novobiocin, whereas it showed classic Michaelis-Menten saturation behavior with UDP-glucose and fidaxomicin (Fig. 4, *A*–*C*). We were unable to achieve full saturation with salinomycin at the maximum concentration tested (100 mM), and kinetic parameters are extrapolated from the fit to the curve (Fig. 4*D*). Kinetic constants for each substrate tested are listed in Table 2.

**Table 2:** Steady state kinetic constants for Ngt-1

Substrate	K_M_ (μM)	*k*_*cat*_ (s^-1^)	*k*_*cat*_/K_M_ (s^-1^M^-1^)
Novobiocin	1.51 ± 0.38 × 10^2^	3.96 ± 0.37 × 10^3^	2.63 × 10^7^
UDP-glucose	1.32 ± 0.13 × 10^2^	3.29 ± 0.06 × 10^3^	2.50 × 10^7^
Fidaxomicin	9.30 ± 2.61 × 10^-1^	2.92 ± 0.43 × 10^2^	3.24 × 10^8^
Salinomycin	1.29 ± 0.69 × 10^2^	1.80 ± 0.63 × 10^3^	1.40 × 10^7^

### Crystallization and structural determination of Ngt-1 complex with novobiocin and UDP

To gain insight into how novobiocin interacts with the Ngt-1 glycosyltransferase, we solved the co-crystal structure of Ngt-1 bound to its substrates. Ngt-1 protein incubated with novobiocin and UDP crystallized in space group *P* 2_1_2_1_2_1_, containing one molecule per asymmetric unit. The X-ray crystal structure was refined to a resolution of 2.0 Å with R_work_ and R_free_ values of 0.1738 and 0.2052, respectively. The electron density map enabled modeling of residues Met1 to Tyr398, except for three flexible loop regions (residues 67–73, 140–152, and 215–228) (Table S4).

Ngt-1 adopts a typical ‘GT-B’ fold characteristic, displaying β/α/β Rossmann-like domains, consistent with CAZy GT-1 family of glycosyltransferases (26). The co-crystal structure revealed three novobiocin molecules bound within the active site and at peripheral regions of the protein (Fig. S3). Attempts to obtain crystals at lower novobiocin concentrations were unsuccessful. The presence of multiple novobiocin molecules is therefore interpreted as a crystallization artifact, likely contributing to crystal packing by stabilizing and bridging neighboring protein molecules. At higher concentrations, novobiocin induces a substrate-inhibited state in Ngt-1 (Fig. 4*A*), and the crystal structure may represent this inhibited conformation (Fig. S4).

**Figure 5:**
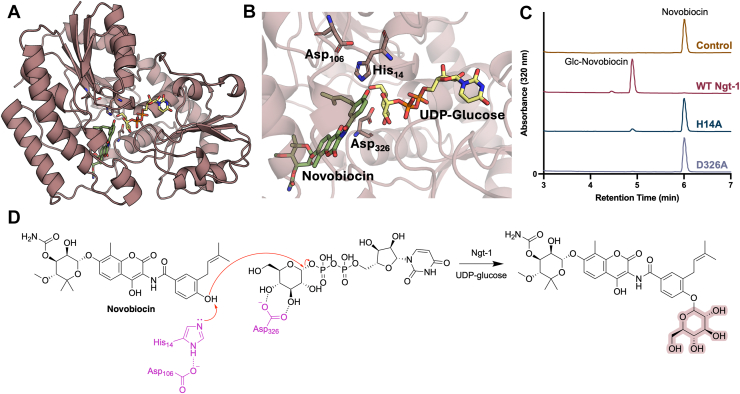
**Structural analysis of Ngt-1.***A*, chai discovery model of Ngt-1 in complex with novobiocin and UDP. *B*, atalytic site of Ngt-1 with His14, Asp106, and Asp326. *C*, catalytic mutants of Ngt-1 result in a loss of novobiocin-modifying activity. *D*, proposed mechanism of novobiocin glycosylation.

Using the AlphaFold-predicted structure of Ngt-1, novobiocin and UDP-glucose were modeled in the Chai Discovery platform (27), yielding a predicted complex highly similar to the experimentally determined crystal structure (Fig. 5, *A* and *B*). The model achieved a high aggregated confidence score of 0.894 (pTM = 0.933, ipTM = 0.884). Structural alignment of the predicted and crystal structures yielded an RMSD of 0.653 Å, with the position of UDP-glucose aligning closely with that observed in the crystal structure (Fig. S4). The predicted binding orientation of novobiocin was aligned with our experimentally determined biochemical assay data.

Analysis of the structure revealed defined binding sites for novobiocin and UDP-glucose and suggested His14, Asp106, and Asp326 serve as key catalytic residues (Fig. 5*B*). Asp106 is predicted to stabilize His14, ensuring proper catalytic orientation. His14 acts as a catalytic base, removing the phenolic proton and thereby facilitating phenolate nucleophilicity. The resulting nucleophile attacks the glucose group of UDP-glucose, which is held in place by Asp326. The functional importance of the residues in direct contact with the substrates (His14 and Asp326) was validated by site-directed mutagenesis. Ngt-1 variants carrying His14Ala and Asp326Ala mutations showed no detectable glycosylated novobiocin production by RP-HPLC (Fig. 5*C*), supporting essential roles for these residues in the proposed mechanism of novobiocin glycosylation (Fig. 5*D*).

A structural homology search using DALI, which identifies proteins with similar three-dimensional folds independent of sequence similarity, revealed Ngt-1 paralogs widespread across *Bacillus* species (Fig. S5) with *Bacillus subtilis* glycosyltransferases Bs-YjiC (28) and Bs-YojK (29) as top two homologs. Bs-YjiC (locus tag: HIR76_20730; accession: CP051860.2) and Bs-YojK (locus tag: HIR76_16890; accession: CP051860.2) are annotated GT-B fold glycosyltransferases encoded within the *B. subtilis* genome. To assess whether related GT-B enzymes confer resistance to novobiocin, the corresponding genes (*yjiC* and *yojK*) were cloned into the hypersensitive *E. coli* BW25113 *ΔbamB ΔtolC* strain. Expression of these homologs resulted in only modest increases in MIC relative to *ngt-1* (Table 3), suggesting that although these enzymes share structural similarity, Ngt-1 possesses enhanced or more specialized activity toward antibiotic modification. Sequence comparison indicated that Ngt-1 shares 45.8% identity with Bs-YjiC and 54.2% with Bs-YojK, while retaining conserved residues characteristic of GT-B glycosyltransferases, supporting its classification within this enzyme family.

**Table 3:** Comparison of novobiocin resistance confered by different *Bacillus* glycosyltransferases

Gene	MIC (μ g/ml)	Fold-increase compared to empty vector
*E. coli ΔbamB ΔtolC*	0.5	-
*E. coli ΔbamB ΔtolC +ngt-1*	128	256-fold
*E. coli ΔbamB ΔtolC +yjiC*	4	8-fold
*E. coli ΔbamB ΔtolC +yojK*	1	2-fold

## Discussion

The environmental resistome encompasses a vast and relatively unexplored reservoir of antibiotic resistance genes (5). Shaped over millennia through microbial competition, these elements encompass a broad range of genetically and mechanistically diverse resistance strategies, many of which are not yet found in clinical settings (4, 5, 7). There is growing evidence that, under the right selective pressures, resistance genes from non-producing environmental bacteria can be mobilized into pathogens (4, 7, 15). This trend highlights the importance of proactive surveillance of environmental microbes and metagenomes to anticipate future threats to antibiotic efficacy.

In this study, we describe the identification and characterization of a novel broad-spectrum glycosyltransferase, Ngt-1, from an environmental *B. thuringiensis* strain. The precise physiological role of Ngt-1 in *B. thuringiensis* remains unclear. RT-qPCR analysis indicated that *ngt-1* expression was not induced by novobiocin exposure. Nevertheless, Ngt-1 catalyzes the inactivation of antibiotics from three chemically and mechanistically distinct classes: aminocoumarins (novobiocin), tiacumicins (fidaxomicin), and polyethers (salinomycin). Together, these findings support the hypothesis that Ngt-1 represents a proto-resistance element-defined as a gene not presently functioning in resistance but possessing the capacity to evolve into a clinically relevant resistance determinant (14).

Ngt-1 belongs to the GT-B family of glycosyltransferases – enzymes traditionally associated with catalyzing the transfer of activated sugars to a variety of acceptor molecules as part of carbohydrate biosynthesis (30, 31). While glycosyltransferases involved in antibiotic resistance – Rgt1438 (rifamycins) and GimA (macrolides) (32, 33) – have been reported, examples demonstrating this degree of substrate promiscuity toward diverse antibiotic scaffolds remain limited. This broadened substrate scope may arise from the inherent flexibility of the N-terminal domain of GT-B glycosyltransferases (26). Although not all the antibiotics evaluated in this study are not all widely used in current clinical practice, they represent structurally diverse scaffolds spanning multiple antibiotic classes. As such, the observed substrate promiscuity of Ngt-1 highlights its potential to act on a broader range of compounds, including those with greater clinical relevance. These findings underscore the importance of investigating environmental resistance enzymes as potential precursors to mechanisms that may emerge under future selective pressures.

Like macrolide (34) and glycopeptide (35) glycosyltransferases, important in antibiotic resistance and biosynthesis, respectively, Ngt-1 adopts the classic two-domain GT-B fold with the enzyme active site at the intersection of the domains. Although novobiocin was found bound to the peripheral sites, this is interpreted as a crystallization artifact. The stacking of novobiocin molecules likely influences their orientation, rendering this arrangement unrepresentative of the catalytically active state. Crystals were only obtained at higher concentrations of novobiocin, suggesting that novobiocin contributes to crystal packing by stabilizing and bridging neighboring protein molecules. Additionally, steady state kinetic analyses revealed a substrate-inhibited state of Ngt-1 at elevated novobiocin concentrations, which may correspond to the state captured in the crystal structure. In contrast, the Chai Discovery-predicted model shows the benzamide group of novobiocin in close proximity to the glucose moiety of UDP-glucose. This arrangement is consistent with the MS/MS and NMR data, demonstrating glycosylation of novobiocin at the benzamide hydroxyl.

We observed limited novobiocin resistance conferred by the homologous *Bacillus* glycosyltransferases Bs-YjiC and Bs-YojK. Glycosyltransferase belonging to this family possesses a flexible N-terminal domain and a rigid C-terminal domain, which may explain the broad acceptor spectrum exhibited by Ngt-1 (26, 28, 29). This is attributed to their Rossmann-like domains, which are found in various metabolic enzymes, allowing them to bind diverse ligands (26). Despite significant sequence similarity, the Bs-GTs did not exhibit the same level of resistance as Ngt-1, suggesting that specific structural features may account for Ngt-1's enhanced activity. These findings raise important questions about the mutational or regulatory changes that could convert such homologs into fully functional resistance genes – an essential hallmark of proto-resistance.

This study focuses on the importance of identifying and functionally characterizing environmental resistance genes before they become a threat in clinical settings. Ngt-1 is a rare example of a functionally novel, broad-spectrum resistance enzyme capable of modifying multiple structurally distinct antibiotics, suggesting a degree of substrate promiscuity that may contribute to low-level or emergent resistance phenotypes. Continued structural and functional studies of GT-B enzymes, such as Ngt-1, can provide deeper insight into how enzymatic flexibility contributes to resistance and how selective pressure in microbial communities shapes their evolution. These findings expand our understanding of the functional diversity and evolutionary potential of antibiotic-modifying glycosyltransferases. Ultimately, understanding the genetic diversity and dynamics of resistance in the environment is critical for anticipating and mitigating future antimicrobial resistance threats.

### Experimental procedures

#### Resistance screen

A soil sample was collected from an agricultural site in Nigeria (NG-24 obtained from our collaborator, Dr O. Ejim). From 1 g of soil, 262 bacterial isolates were obtained from morphologically distinct colonies. Minimum inhibitory concentrations (MICs) for each isolate were determined against a panel of 60 antimicrobial agents as part of a large-scale phenotypic resistance screen to assess antibiotic susceptibility.

### Genomic DNA extraction and genome sequencing

**WAC10774 A:** Promega Wizard Genomic DNA Purification Kit [REF: A1125] was used for extracting genomic DNA from the *Amycalotopsis* strain. The strain was cultured for 3 days in Tryptic Soy Broth with aeration in a 30 °C shaking incubator (250 rpm). The gDNA was sequenced using both Illumina and nanopore sequencing, with PacBio providing long- and short-reads. NCBI Accession number: SAMN57036074.

**WAC10774 B:** *B. thuringiensis* cells were cultured overnight in 3 ml of Tryptic Soy Broth at 37 °C with shaking at 250 rpm, then collected by centrifugation at 12,000 rpm for 5 min. To lyse the cells, 150 mg of 0.1 mm beads were added and vortexed vigorously for 2 min. Further lysis was performed by adding 400 μl of SET buffer (75 mM NaCl, 25 mM EDTA, 20 mM Tris HCl pH 7.5) with 3 mg/ml lysozyme, followed by incubation at 37 °C for 60 min. Then, 0.1 volumes of 10% SDS and 0.1 volumes of 1 mg/ml proteinase K were added, and incubation continued at 56 °C for 30 min. The suspension was transferred to a 2 ml tube to separate the beads. DNA was extracted by adding 500 μl of phenol:chloroform alcohol, vortexing for 20 s, and centrifuging at maximum speed for 10 min at 4 °C. The supernatant was transferred to a fresh tube, and this extraction was repeated three times. To precipitate the DNA, 0.1 volumes of 3 M sodium acetate (pH 5.2) and 2.5 volumes of ice-cold ethanol were added, mixed, and incubated at −20 °C overnight. The DNA was pelleted by centrifugation at maximum speed for 30 min at 4 °C, and the supernatant was discarded. The pellet was washed with 0.5 ml of 70% ethanol, centrifuged at 16,000*g* for 10 min at 4 °C, and the supernatant was discarded. This wash was repeated, and the pellet was resuspended in 250 μl of TE buffer. The genomic DNA was sequenced using Oxford Nanopore (Plasmidsaurus). NCBI Accession number: SAMN57016808.

#### HPLC methodology

HPLC was employed using an Agilent 1260 Infinity II analytical LC system to assess the inactivation of novobiocin, based on the appearance of the modified product peak. Water with 0.1% formic acid (solvent A) and acetonitrile with 0.1% formic acid (solvent B) served as the mobile phases, and an XSelect CSH C18 5 μm (4.5 × 100 mm) column was selected as the stationary phase. Gradient elution started with 95% solution A and 5% solution B over 10 min, then changed to 5% solution A and 95% solution B at the end of 10 min, with a flow rate of 1.0 ml/min. The final concentrations were maintained for 1 min, then the gradient was reverted to 95% solution A and 5% solution B over 1 min and maintained for another 4 min to flush remaining compounds from the column. A 3-min post-run period was used to equilibrate the pumps. Novobiocin was detected using UV at 320 nm.

#### Antibiotic inactivation assay

*B. thuringiensis* WAC10774 B cells were harvested by centrifugation and resuspended in 50 mM Hepes buffer (pH 7.5). To test for inactivation, the suspension was supplemented with 50 μM novobiocin and incubated at 37 °C for 30 min. For cell-free extracts, 250 μM UDP-glucose was added as a co-substrate. After incubation, equal volumes of methanol were added to quench the reaction. The samples were then centrifuged for 2 min at maximum speed, and 10 μl of the supernatant was analyzed by HPLC. Novobiocin inactivation was indicated by the disappearance of its peak and the appearance of a new hydrophilic product peak.

#### Characterization of modified-antibiotic products

Mass analysis was conducted to characterize the modified novobiocin, fidaxomicin, and salinomycin products using LC-QTOF-MS on an Agilent 6545 LC-Q-TOF. Water with 0.1% formic acid (solution A) and acetonitrile with 0.1% formic acid (solution B) served as the mobile phases. An Agilent Eclipse XDB-C8 column (3.5 μm, 2.1 × 100 mm) was used as the stationary phase. Gradient elution started with 95% solution A and 5% solution B over 8 min, ending at 5% solution A and 95% solution B at a flow rate of 0.4 ml/min. The LC-QTOF-MS was operated in positive mode to assess the mass of all relevant peaks. For fragmentation pattern analysis, targeted MS/MS was applied, with fragmentation energies set to 10 V and 15 V for novobiocin and its modified product, respectively.

#### Purification of NGT from *B. thuringiensis*

*B. thuringiensis* cells, grown under the conditions specified above, were collected by centrifugation at 6000 rpm for 15 min from a 2 L liquid culture. The collected cells were washed and resuspended in 100 ml of 50 mM Hepes with 1 mM EDTA at pH 7.5 and kept on ice. Two Pierce protease inhibitor (EDTA-free) tablets, bovine pancreas deoxyribonuclease I, and egg white lysozyme were added to the resuspended sample. The sample was processed through a continuous-flow cell disruptor (Constant Systems) at 40 PSI (approximately 20 kPa) twice to lyse the cells. The lysate was clarified by centrifuging at 16,000 rpm for 15 min. Novobiocin inactivation activity was assessed using the HPLC method described above.

Total protein in the crude cell-free extract was sequentially precipitated using ammonium sulfate at w/v concentrations of 20%, 40%, 60%, and 80%. The sample was incubated at 4 °C with each cutoff for 1 to 2 h. Proteins were precipitated, collected by centrifugation, and resuspended in 50 mM Hepes + 1 mM EDTA pH 7.5 buffer. Protein obtained from the 60% ammonium sulfate cut was resuspended in 10 ml buffer. The enzyme solution was then applied to a Phenyl Sepharose 6 Fast Flow (GE Healthcare BioScience) column, equilibrated with 1 M ammonium sulfate, 25 mM Hepes, 1 mM EDTA, pH 7.5. The column was washed with the equilibration buffer, and the enzyme was manually eluted with a step gradient using buffers containing 0.4 M, 0.2 M, 0.1 M, and 0 M ammonium sulfate. Active fractions were pooled and concentrated using filter centrifugation. The concentrated active sample was desalted by dialysis against 25 mM Hepes + 1 mM EDTA (3× 2 L, 1 h each).

The enzyme solution (6 ml) from the previous step was applied to a Hydroxyapatite Bio-Gel HT Gel column equilibrated with 10 mM NaPO_4_, pH 7.5. After washing the column with the equilibration buffer, the enzyme was eluted manually using a step gradient from 10 mM NaPO_4_ to 0.4 mM NaPO_4_. Enzyme activity was measured, and the active fractions were pooled (15 ml). The sample was adjusted to 5% glycerol to promote crowding. The active sample was desalted by dialysis against 25 mM Hepes + 1 mM EDTA + 5% glycerol (3 times, 2 L each, 1 h per step).

The enzyme solution (14 ml) was applied to a HiTrap Q HP 5 ml column pre-equilibrated with 25 mM Hepes + 1 mM EDTA + 5% glycerol buffer at pH 7.5. The column was washed with the equilibration buffer, and the enzyme was eluted using a linear gradient from 25 mM Hepes + 1 mM EDTA + 5% glycerol to 1 M NaCl in the same buffer. The flow rate was 5 ml/min, and 1.8 ml fractions were collected for enzyme assay.

#### Proteomic analysis

Protein in the final semi-pure active sample (purified from *B. thuringiensis* crude sample) were precipitated with acetone (4:1) and resuspended in denaturing buffer (100 mM ammonium bicarbonate + 8 M Urea). The protein sample was reduced with 5 mM TCEP and alkylated with 10 mM iodoacetamide. The reaction was quenched with 12 mM N-acetyl cysteine. Final digestion was performed overnight using 10 μg of sequencing-grade trypsin. Digested samples were cleaned with a C18 Sep-Pak cartridge, and peptides were eluted with 100% acetonitrile + 0.1% FA. The peptide samples were dried using a vacuum centrifuge (Thermo Fisher Scientific Savant SpeedVac SPD120 Vacuum Concentrator) and resuspended in 50 μl of Milli-Q water with 0.1% formic acid.

Peptide mass spectrometry was conducted using the Agilent 6545 LC-Q-TOF system with an Agilent ZORBAX 300SB-C18 column (5 μm, 2.1 × 150 mm) as the stationary phase. Water with 0.1% formic acid (solution A) and acetonitrile with 0.1% formic acid (solution B) served as the mobile phases. Gradient elution started with 98% of solution A and 2% of solution B over 38 min, ending at 5% solution A and 95% solution B with a flow rate of 0.4 ml/min. The LC-QTOF-MS operated in positive mode to analyze the mass of relevant peaks. For fragmentation pattern analysis, DIA auto MS/MS was used, with a mass range of 100 to 1700*m/z*. The peptide data were analyzed with MSFragger, a high-speed database search tool integrated into FragPipe software (v 23.1) (https://fragpipe.nesvilab.org, https://github.com/Nesvilab/FragPipe), which identifies peptides based on the mass spectrometry data (24). Peptide mapping involved comparing the identified peptides to the *B. thuringiensis* proteome (UP000011719: *B. thuringiensis serovar thuringiensis* str. IS5056) to predict the proteins present.

#### Over-expression and purification of NGT enzyme

To overexpress NGT, the gene was amplified from the genome of the *B. thuringiensis* strain using PCR with the forward primer: CGCGTACTCGAGATGGCAAATGTACTCGTAATAAAT and the reverse primer: CGCCATGGATCCGTTAATCTTTACATACGGCTTCAT. The GT gene was then ligated into pET28a using restriction sites NheI and XhoI, transformed into *E. coli* Top 10, and plated on LB Kanamycin (50 μg/ml) plates. Positive colonies were validated through colony PCR, plasmids were isolated, and the constructs were electroporated into *E. coli* BL21 (DE3). *The ngt-1* gene was overexpressed in *E. coli* BL21 (DE3) using 0.5 mM IPTG at 18 °C overnight. Cells were harvested by centrifugation at 6500*g* for 30 min and stored at −20 °C. For cell lysis, the cell pellet was resuspended in buffer A (50 mM Hepes pH 8.0, 300 mM NaCl, 5 mM imidazole, 1 mM DTT), to which Pierce protease inhibitor (EDTA-free) tablets and DNase were added. The cells were lysed using a continuous-flow cell disruptor (Constant Systems) at 40 PSI (approximately 20 kPa). The lysate was clarified by centrifugation at 20,000*g* for 30 min, then incubated with Ni-NTA agarose (Qiagen) pre-equilibrated with buffer A at 4 °C. The resin was transferred to a chromatography column and washed with two column volumes of buffer A. Proteins were eluted with buffer B (50 mM Hepes pH 8.0, 300 mM NaCl, 250 mM imidazole, 1 mM DTT). Fractions containing purified GT enzyme were buffer-exchanged using the AKTA Pure system and a HiLoad 16/600 Superdex 200 PG size-exclusion column against buffer (25 mM Hepes pH 7.5, 150 mM NaCl, 1 mM DTT). The purified GT enzyme samples were pooled and concentrated.

#### Antibiotic susceptibility testing

For susceptibility testing, MICs were measured for novobiocin and glucosylated-novobiocin, along with fidaxomicin and salinomycin, in Cation-adjusted Mueller-Hinton Broth using the CLSI standard broth microdilution method. This was performed with the hyperpermeable and efflux-deficient *E. coli* strain BW25113 Δ*bamB* Δ*tolC* (*E. coli* ΔΔ) (23). To confirm novobiocin resistance, the *ngt-1* gene was cloned into the sensitive *E. coli* ΔΔ strain using our in-house pGDP1 vector. MICs were then compared between *E. coli* ΔΔ and *E. coli* ΔΔ +*ngt-1* to evaluate the increase in resistance. The inoculum was prepared *via* the direct colony suspension method, with a final OD_600_ of 0.001. Plates were incubated overnight at 37 °C and measured with a Synergy H1 microplate reader.

#### RT-qPCR

Bacterial cultures of *B. thuringiensis* were grown to mid-log phase and incubated with increasing concentrations of novobiocin (0, 0.0325, 00625, 0.125, 0.25 μg/ml) for 2 h. 1.5 ml of cell culture was centrifuged, harvested, and stored at −80 °C. RNA extraction was performed using the QIAGEN RNeasy kit according to its protocol. cDNA synthesis was carried out using Maxima H Minus cDNA synthesis master mix with dsDNase (catalog number M1681, Thermo Fisher Scientific). cDNA (0.5 μg) was quantified on 96-well optical microplates using PowerUp SYBR Green Master Mix for qPCR (catalog number A25742, Applied Biosystems) with 2.25 μM of each of the forward and reverse primers. Assays included at least two biological replicates and two technical replicates per biological replicate. The assay also included a housekeeping gene (*gatB*) and appropriate controls (positive control with gDNA, no reverse transcriptase control, no template control).

#### Steady-state kinetics

A pyruvate kinase lactate dehydrogenase coupled assay was developed to evaluate the steady-state kinetic properties of Ngt-1. In this assay, the UDP produced by the enzyme is coupled to NADH oxidation *via* pyruvate kinase and lactate dehydrogenase (LDH) (25). The decrease in absorbance at 340 nm correlates with the amount of glucosylated product formed. All kinetic assays were performed in 96-well plates with a final reaction volume of 100 μl, containing 50 mM Hepes (pH 7.5), 40 mM KCl, and 10 mM MgCl_2_ with 1 μM GT enzyme. Reactions were initiated by adding UDP-glucose. Measurements were recorded on the BioTek Synergy Neo2 Multimode Reader. Three independent replicates were conducted at each substrate concentration. The kinetic data were fitted to the Michaelis–Menten model for UDP-glucose and fidaxomicin, and to the Substrate Inhibition model for novobiocin, using GraphPad Prism (www.graphpad.com).

#### Site-directed mutagenesis

Amino acid residues His14 and Asp326 were mutated to confirm their roles as catalytic residues. Site-directed mutagenesis was performed using a standard kinase, ligase, and DpnI enzyme mix for the mutagenesis protocol. The point mutations were introduced at the 5′ end of the forward primer to create H14 A, H14 N, D326 A, and D326 N variants. The PCR product was then used in the Kinase Ligase DpnI enzyme mix reaction: 1 μl PNK, 1 μl T4 ligase, 1.5 μl ligase buffer, 5 μl PCR product, topped up with nuclease-free water to reach a total volume of 15 μl. This reaction was incubated at room temperature for 1.5 h, followed by DpnI digestion with 1 μl DpnI and 4 μl Cutsmart added to the ligation mixture. The total reaction volume was then adjusted to 40 μl with nuclease-free water. The mixture was further incubated at 37 °C for 1 to 2 h before transformation into *E. coli* BL21.

#### Crystallization and X-ray structure of Ngt-1

A protein sample at 10 mg/ml was incubated with novobiocin (5 mM) and UDP (4 mM) and used for initial crystallization screens with four commercial kits from Hampton Research using the sitting-drop vapor diffusion method. All experiments were performed at 20 °C. Conditions that yielded crystals were reproduced with in-house buffers and further optimized for pH, protein concentration, precipitant, and drop volume. The best crystals of Ngt-1 in complex with its substrates were obtained by mixing 2 μl of protein (8 mg/ml) with 2 μl of optimized buffer containing 0.16 mM magnesium acetate, 0.08 mM Na-cacodylate at pH 6.7, 16% PEG 8000 (w/v), and 10% glycerol (v/v). Initial needle-like crystals were further optimized into rectangular box-shaped crystals. Crystal formation occurred within 24 h. X-ray data were collected at McMaster University using a Rigaku BioSAXS-1000 combined with a MicroMax-007 HF X-ray system. Diffraction data were processed and scaled with CrysAlis^PRO^ by Rigaku Oxford Diffraction. Data were processed to a 2.0 Å resolution in the space group 21 21 21, with cell dimensions of a = 54.0292 Å, b = 95.2194 Å, c = 102.9 Å. The phase problem was solved by molecular replacement using Phaser-MR from the PHENIX suite (36), employing the AlphaFold-predicted structure of Ngt-1 as a template. The structure was refined through iterative manual model building with COOT (37) and refinement with PHENIX (36). Structural figures were generated with PyMOL.

#### Protein homology search and phylogenetic reconstruction

To identify homologs of the glycosyltransferase protein (227 amino acids), a BLASTP search was conducted against the UniRef90 database using DIAMOND to retrieve the top 100 hits ranked by E-value. The query sequence was included to form a final set of 101 protein sequences. Sequences were aligned with MUSCLE. The resulting alignment was trimmed with trimAl using a gap threshold of 0.05 (−gt 0.05). A maximum likelihood phylogenetic tree was constructed with IQ-TREE 2 (v2.2.2.7) (38). The best-fit substitution model was chosen using ModelFinder (39). Branch support was evaluated with ultrafast bootstrap (UFBoot2) (40) with 1000 replicates. The phylogenetic tree was visualized using iTOL (41).

## Data availability

Whole genome sequences of *Amycolatopsis* sp. (WAC10774 A) and *B. thuringiensis* (WAC10774 B) are deposited on NCBI under accession codes SAMN57036074 and SAMN57016808 respectively.

The X-ray crystal structure for Ngt-1 is deposited on the Protein Data Bank with PDB ID: 10ZG/pdb_000010 zg.

NMR data is deposited on Biological MagneticResonance Data Bank with Entry ID: BMRbig139, Title: Novobiocin-Glu, and Entry https://doi.org/10.13018/bmrbig139.

Peptide mass spectrometry data for proteomics studies is deposited under the open-access repository Figshare under https://doi.org/10.6084/m9.figshare.31972497 URL: https://doi.org/10.6084/m9.figshare.31972497.

## Supporting information

This article contains supporting information.

## Conflict of interest

The authors declare that they have no conflicts of interest with the contents of this article.
