# Supplementary material for: Ngt-1: A glycosyltransferase that confers resistance to three distinct antibiotic classes
Source: J Biol Chem. 2026 Apr 22;302(6):111479. doi: 10.1016/j.jbc.2026.111479 (PMC13214531; doi:10.1016/j.jbc.2026.111479)
Supplement: Supplementary Figures and Tables [file mmc1.docx]

**Ngt-1: a glycosyltransferase that confers resistance to three distinct antibiotic classes.**

Amna Abbas^1^, David Sychantha^1,2^, Kalinka Koteva^1^, Mei Chiao^1^, Akosiererem Sokaribo^1^, Dirk Hackenberger^1^, Sara Andres^1^, Gerard D. Wright^1,*^.

**Supporting Information:**


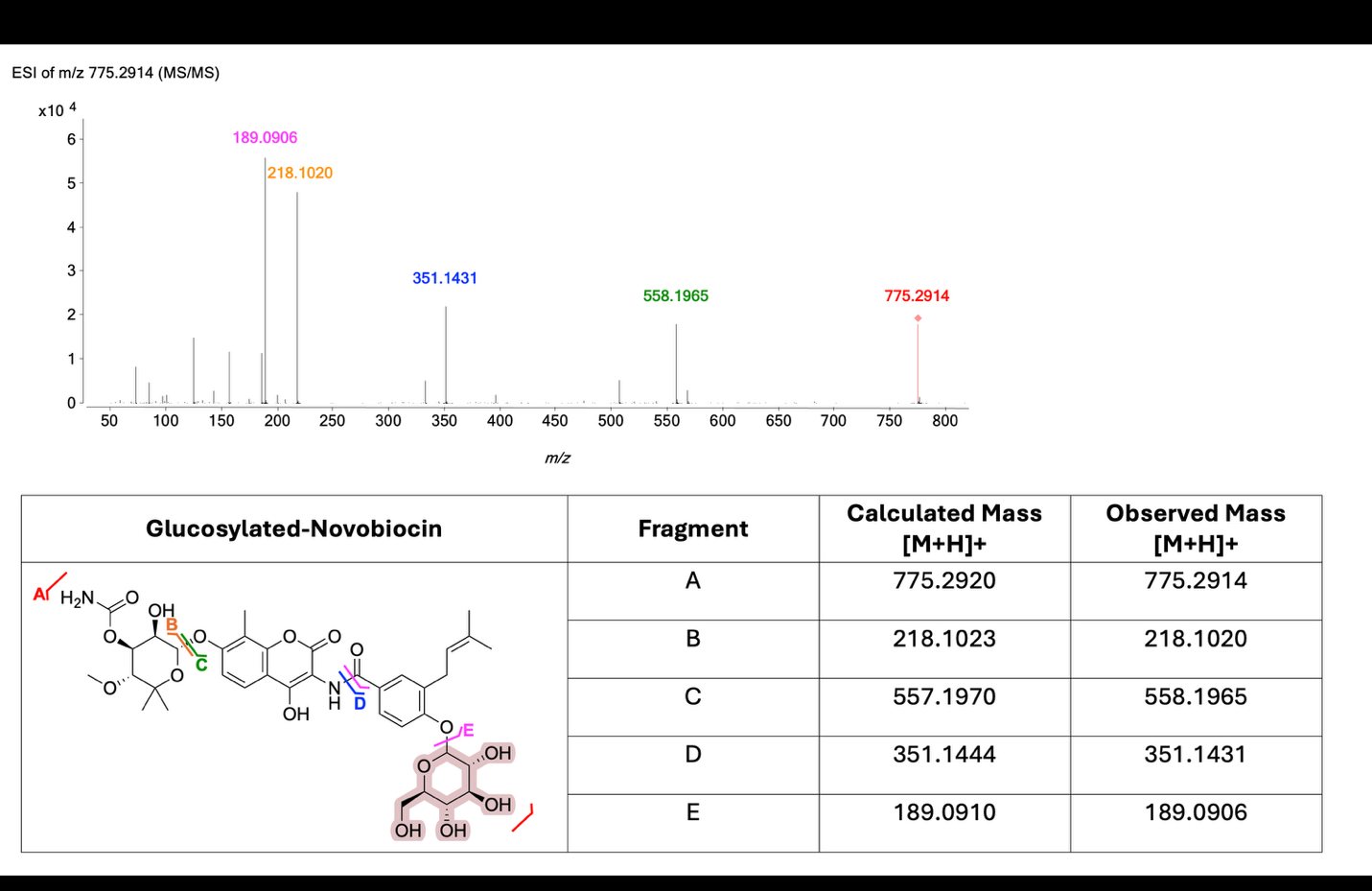


**Figure S1:** ESI-MS/MS of 775.29 [M+H]^+^ glycosylated-novobiocin (bottom) reveal the site of modification to be at the benzamide hydroxyl group, corresponding to the 351.1431 *m/z* peak observed.

**Table S1**: Complete ^1^H and ^13^C assignments of glycosylated novobiocin, compared to novobiocin in dmso-d6.

| Assignment | Novobiocin* | | Inactivated product | |
| --- | --- | --- | --- | --- |
|  | ^1^H (ppm, multiplicity, couplings, Hz)) | ^13^C** | ^1^H (ppm, multiplicity, couplings, Hz)) | ^13^C |
| 1 | - | 126 |  | 126.8 |
| 2 | 7.73 (m, 1H) | 129.4 | 7.78 (d, *J* = 2.2 Hz, 1H) | 129.1 |
| 3 | - | 127.2 |  | 127.2 |
| 4 | - | *160* |  | 157.7 |
| **5** | 6.84 (d, *J* = 8.3 Hz, 1H) | 113.9 | 7.15 (dd, *J* = 8.9, 5.9 Hz, 1H) | 113.3 |
| 6 | 7.70 (m, 1H) | 127.2 | 7.83 (dd, *J* = 8.6, 2.3 Hz, 1H) | 126.8 |
| 7 | 3.26 (d, *J* = 7.3 Hz, 2H) | 27.60 | 3.32 (m, 1H)  3.44 (m, 1H) | 28.07 |
| 8 | 5.34 – 5.28 (m, 1H) | 122.3 | 5.34(m, 1H) | 122.3 |
| 9 | - | 126.9 | - | 131.3 |
| 10 | 1.70 (s, 3H) | 17.3 | 1.70 (s, 3H) | 17.6 |
| 11 | 1.70 (s, 3H) | 25.3 | 1.70 (s, 3H) | 25.3 |
| 12 | - | 166 |  | 166.2 |
| 1’ | - | 160.8 |  | 160.6 |
| 2’ | - | 100.7 |  | 101.4 |
| 3’ | - | 160 |  | 163.5 |
| 4’ | 7.73 (m, 1H) | 121.7 | 7.73 (d, *J* = 8.8 Hz, 1H) | 121.7 |
| 5’ | 7.12 (d, *J* = 9.0 Hz, 1H) | 109.5 | 7.15 (dd, *J* = 8.9, 5.9 Hz, 1H) | 109.4 |
| 6’ | - | 155.7 | - | 156.8 |
| 7’ | - | 112.9 |  | 112.9 |
| 8’ | - | 150.7 |  | 150.7 |
| 9’ | - | 112.7 |  | 112.7 |
| 10’ | 2.21 (s, 3H) | 7.94 | 2.22 (s, 3H) | 7.94 |
| 1” | 5.52 (d, *J* = 2.5 Hz, 1H) | 98.2 | 5.53 (d, *J* = 2.5 Hz, 1H) | 98.2 |
| 2” | 4.07 (dt, *J* = 5.5, 2.9 Hz, 1H) | 68.5 | 4.08 (dt, *J* = 5.5, 2.9 Hz, 1H) | 68.5 |
| 3” | 5.16 (dd, *J* = 9.8, 3.2 Hz, 1H) | 70.1 | 5.16 (dd, *J* = 9.8, 3.2 Hz, 1H) | 70.1 |
| 4” | 3.52 – 3.45 (m, 1H) | 80.5 | 3.52 – 3.36 (m, 4H) | 80.7 |
| 5” | - | 77.9 | - | 78.1 |
| 6” | 3.47 (s, 3H) | 60.8 | 3.47 (s, 3H) | 60.8 |
| 7” | 1.27 (s, 3H) | 28.2 | 1.27 (s, 3H) | 28.2 |
| 8” | 1.06 (s, 3H) | 22.4 | 1.06 (s, 3H) | 22.4 |
| 9” | - | 156.6 |  | 156.2 |
| 1’’’ |  |  | 4.94 (d, *J* = 7.0 Hz, 1H) | 100.4 |
| 2’’’ |  |  | 3.31 (m, 1H) | 73.0 |
| 3’’’ |  |  | 3.19 (td, *J* = 8.8, 4.9 Hz, 1H) | 69.5 |
| 4’’’ |  |  | 3.39 (m, 1H) | 76.6 |
| 5’’’ |  |  | 3.30 (m, 1H) | 76.9 |
| 6’’’ |  |  | 3.72 (ddd, *J* = 11.9, 5.2, 2.1 Hz, 1H);  3.47 (m, 1H) | 60.5 |

References: * [**https://doi.org/10.1002/mrc.1260260211**](https://doi.org/10.1002/mrc.1260260211)

**[10.7164/antibiotics.29.710](https://doi.org/10.7164/antibiotics.29.710)

^1^H- NMR spectra of glycosylated novobiocin in dmso-d6

^13^C NMR spectra of glycosylated novobiocin in dmso-d6

^1^H-^1^H-COSY spectra of glycosylated novobiocin in dmso-d6

^1^H-^13^C- HSQC spectra of glycosylated novobiocin in dmso-d6

^1^H-^13^C-HSQC-TOCSY spectra of glycosylated novobiocin in dmso-d6

^1^H-^13^C-HMBC spectra of glycosylated novobiocin in dmso-d6

**Table S2:** Partial purification of glycosyltransferase from *Bacillus thuringiensis* cell culture.


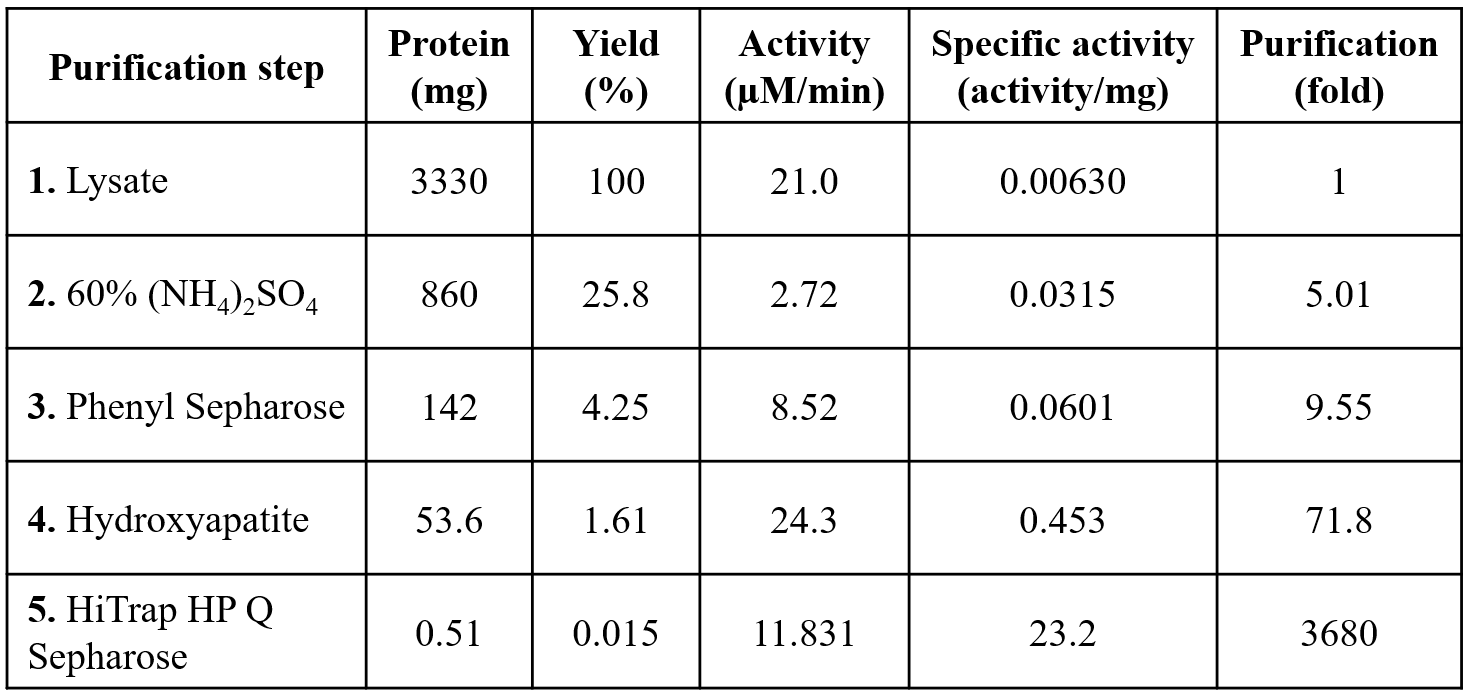


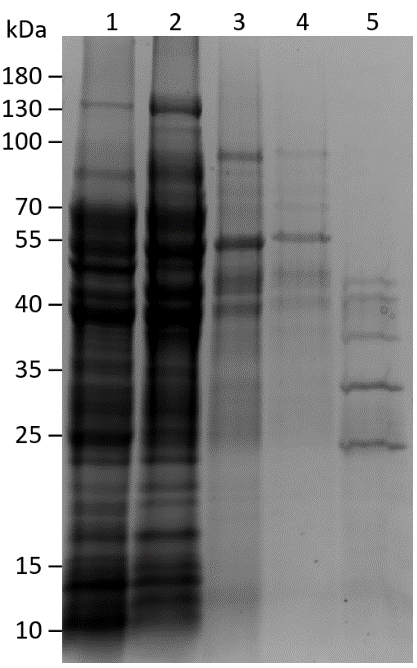


**Figure S2:** SDS–PAGE analysis of active proteins obtained from each purification step. Proteins were visualized by Coomassie blue staining. Lanes labelled as 1, crude cell lysate; 2, 60% ammonium sulphate precipitation; 3, active fractions after hydrophobic interaction chromatography on phenyl-Sepharose; 4, after hydroxyapatite chromatography; and 5, after anion-exchange chromatography on HiTrap HP Q.

**Table S3:** Antibiotics tested against *ngt-1* for resistance.

| **Antibiotic** | **Antibiotic Class** | **Resistance shown by *ngt-1*** |
| --- | --- | --- |
| Novobiocin | Aminocoumarin | Yes |
| Coumermycin A1 | Aminocoumarin | No |
| azithromycin | Macrolide | No |
| erythromycin estolate | Macrolide | No |
| erythromycin hydrate | Macrolide | No |
| clarithromycin | Macrolide | No |
| Rifaximin | Rifamycin | No |
| Rifampicin | Rifamycin | No |
| Rifabutin | Rifamycin | No |
| Rifamycin sv | Rifamycin | No |
| Fidaxomicin | Tiacumicin | Yes |
| Tylosin | Macrolide | No |
| Oxytetracycline | Tetracycline | No |
| Nourseothricin | Aminoglycoside | No |
| Apramycin | Aminoglycoside | No |
| Streptomycin | Aminoglycoside | No |
| Gentamicin | Aminoglycoside | No |
| Spectinomycin | Aminocyclitol | No |
| Salinomycin | Polyether | Yes |

**Table S4:** Ngt-1 crystal structure parameters.

|  | **Ngt-1** |
| --- | --- |
| **Wavelength (Å)** | 1.542 |
| **Resolution range (Å)** | 27.02 – 2.00 (2.07-2.00) |
| **Space group** | P 21 21 21 |
| **Unit cell** | 54.029 95.219 102.9 90 90 90 |
| **Total reflections (#)** | 174485 |
| **Unique reflections (#)** | 36540 |
| **Multiplicity** | 4.8 |
| **Completeness (%)** | 99.9% (99.8%) |
| **Mean I/sigma(I)** | 21 (4.4) |
| **Wilson B-factor** | 23.78 |
| **R-merge** | 0.055 (0.282) |
| **R-meas** | 0.062 (0.316) |
| **R-pim** | 0.028 (0.138) |
| **CC1/2** | 0.999 (0.940) |
| **Reflections used in refinement** | 35055 (2340) |
| **Reflections used for R-free** | 1946 (134) |
| **R-work** | 0.1738 (0.1851) |
| **R-free** | 0.2052 (0.2313) |
| **Number of non-hydrogen atoms** | 3336 |
| **macromolecules** | 2964 |
| **ligands** | 157 |
| **solvent** | 215 |
| **Protein residues** | 370 |
|  |  |
| **RMS(bonds)** | 0.009 |
| **RMS(angles)** | 0.91 |
| **Ramachandran favored (%)** | 97.79 |
| **Ramachandran allowed (%)** | 2.21 |
| **Ramachandran outliers (%)** | 0.00 |
| **Rotamer outliers (%)** | 0.00 |
| **Clashscore** | 2.26 |
| **Average B-factor** | 31.31 |
| **macromolecules** | 30.69 |
| **ligands** | 46.56 |
| **solvent** | 32.08 |


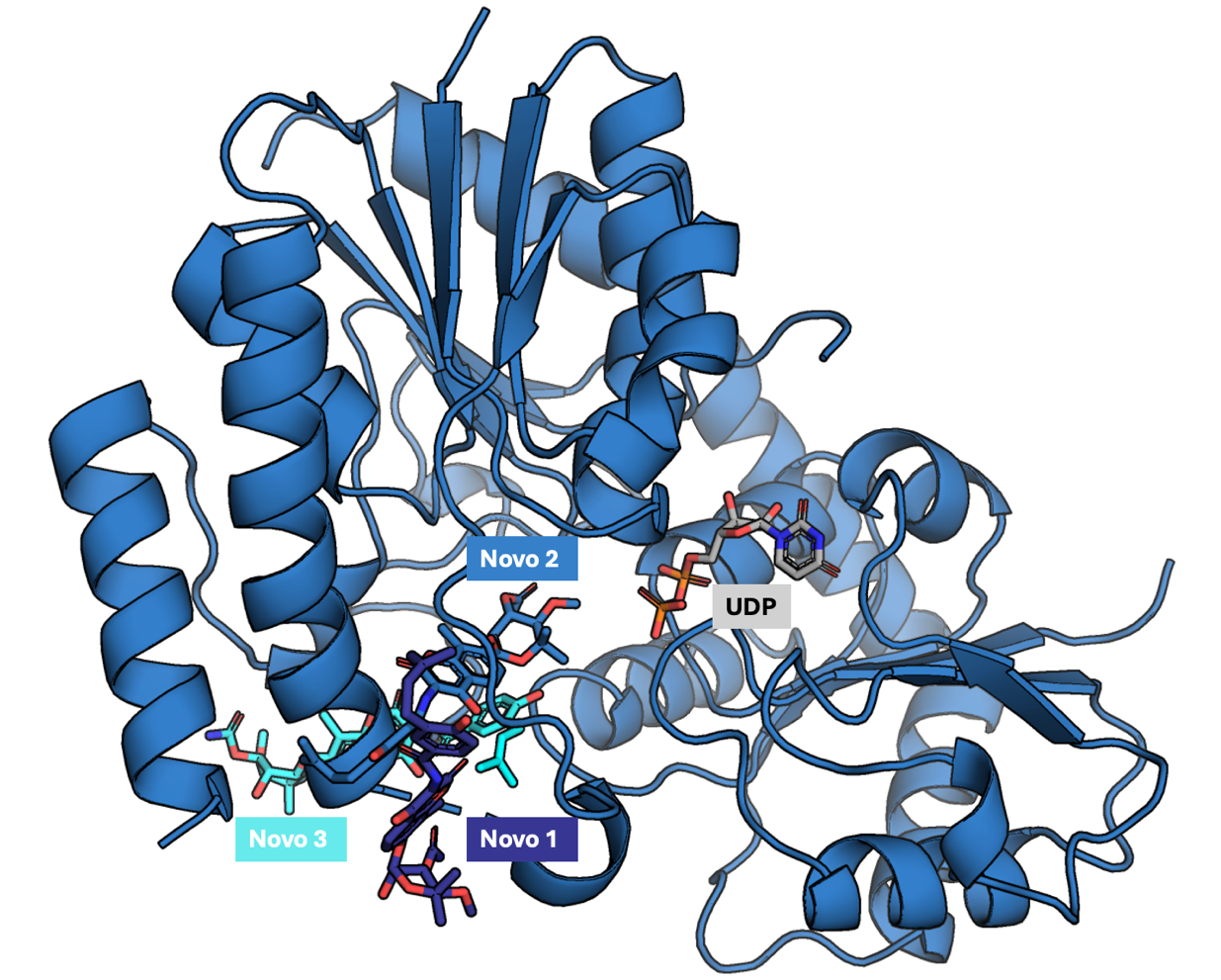


**Figure S3:** Crystal structure of Ngt-1 in complex with novobiocin and UDP.


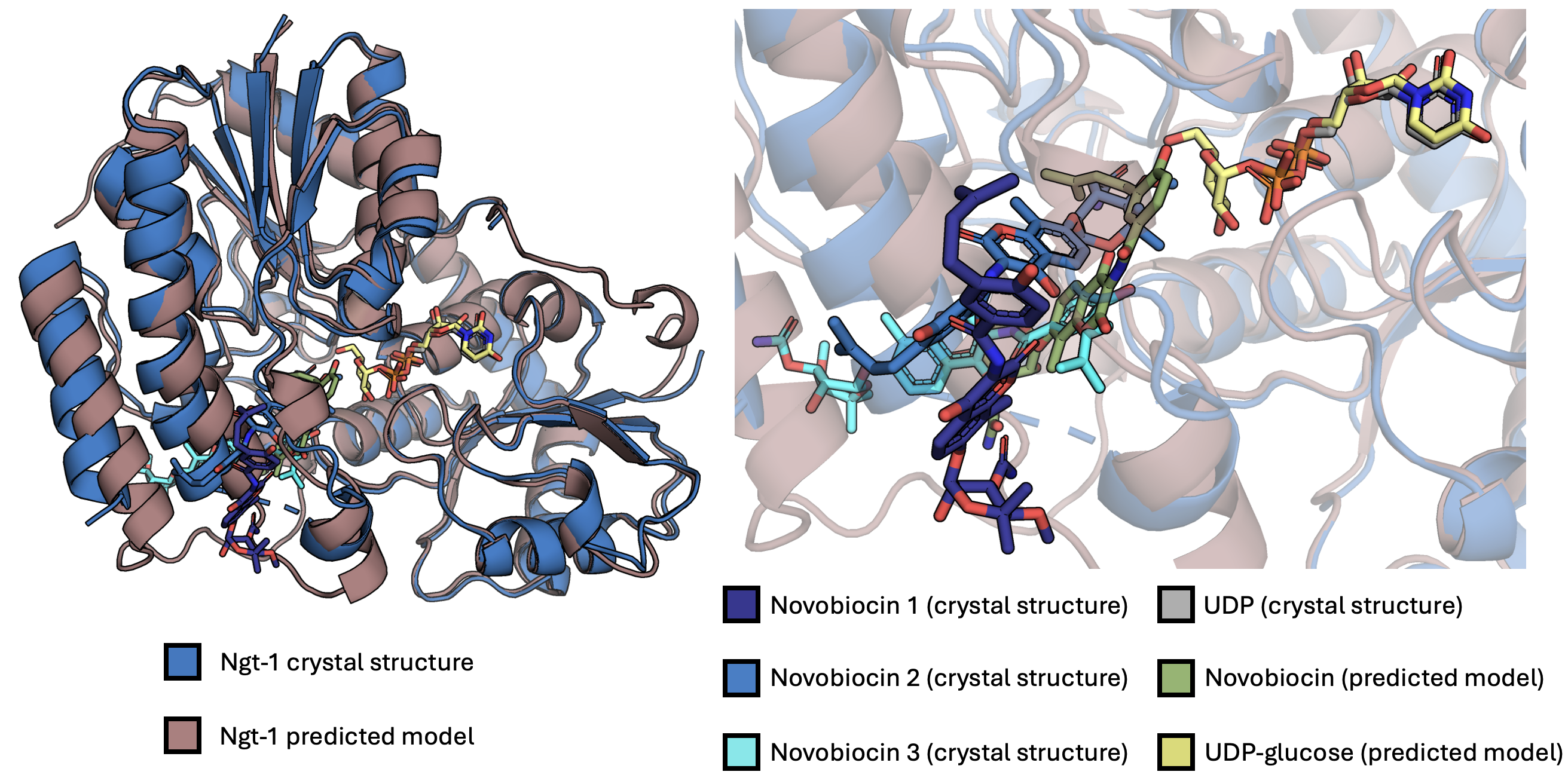


**Figure S4:** Ngt-1 predicted structure (pink) overlapped with crystal structure (blue), giving an RMSD=0.653 Å.


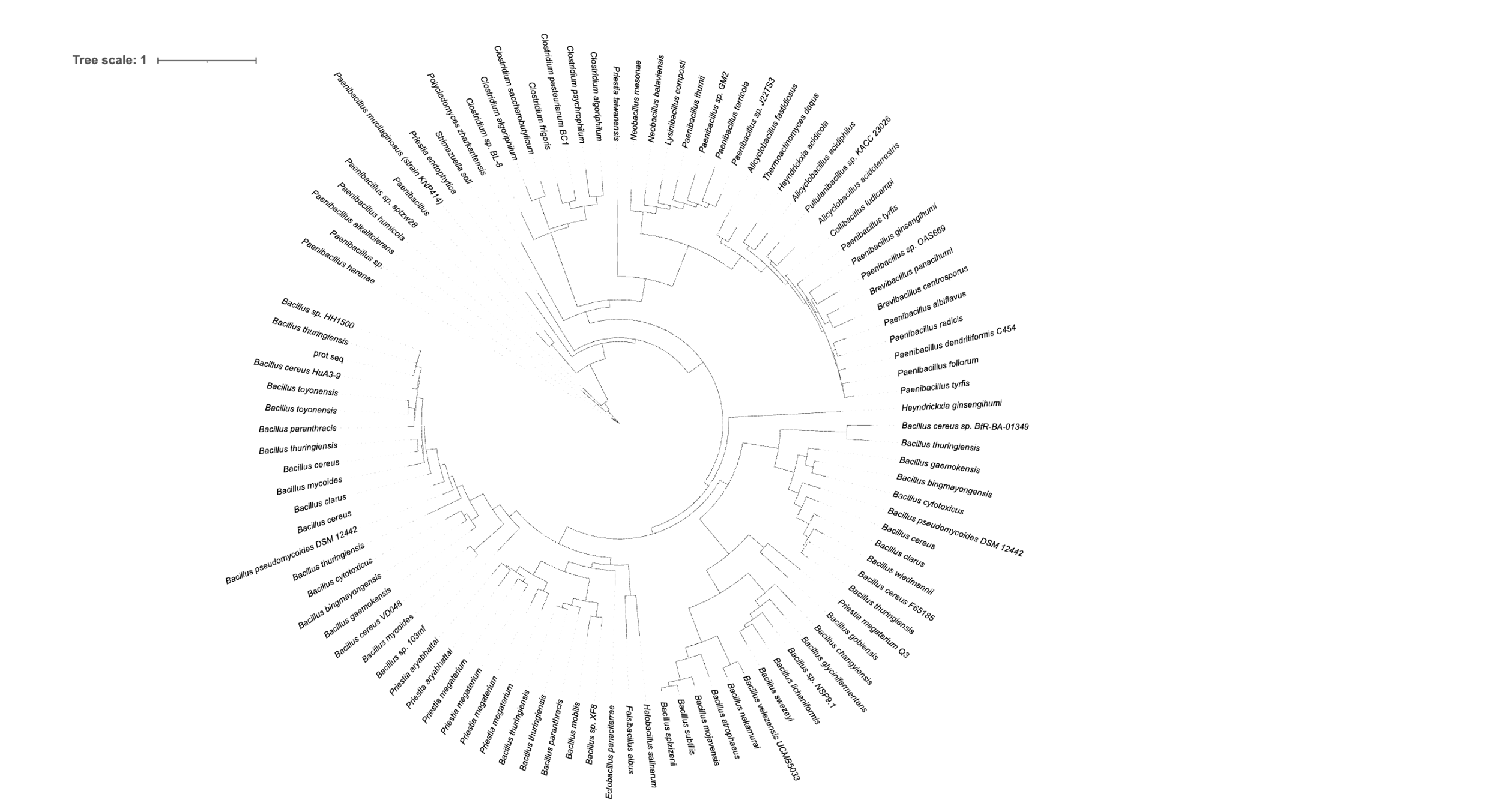


**Figure S5:** Phylogenetic tree showing the distribution of Ngt-1 paralogs across different species, predominantly within *Bacillus***.**
